# Animal models in peritoneal dialysis

**DOI:** 10.3389/fphys.2015.00244

**Published:** 2015-09-01

**Authors:** Olga Nikitidou, Vasiliki I. Peppa, Konstantinos Leivaditis, Theodoros Eleftheriadis, Sotirios G. Zarogiannis, Vassilios Liakopoulos

**Affiliations:** ^1^Division of Nephrology and Hypertension, 1st Department of Internal Medicine, School of Medicine, American Hellenic Educational Progressive Association Hospital, Aristotle University of ThessalonikiThessaloniki, Greece; ^2^Department of Physiology, Faculty of Medicine, BIOPOLIS, University of ThessalyLarissa, Greece

**Keywords:** animal models, ion and water transport, membrane physiology, peritoneal dialysis, ussing chamber

## Abstract

Peritoneal dialysis (PD) has been extensively used over the past years as a method of kidney replacement therapy for patients with end stage renal disease (ESRD). In an attempt to better understand the properties of the peritoneal membrane and the mechanisms involved in major complications associated with PD, such as inflammation, peritonitis and peritoneal injury, both *in vivo* and *ex vivo* animal models have been used. The aim of the present review is to briefly describe the animal models that have been used, and comment on the main problems encountered while working with these models. Moreover, the differences characterizing these animal models, as well as, the differences with humans are highlighted. Finally, it is suggested that the use of standardized protocols is a necessity in order to take full advantage of animal models, extrapolate their results in humans, overcome the problems related to PD and help promote its use.

Peritoneal dialysis (PD) is a well-established method of kidney replacement therapy. It is currently estimated that the end stage renal disease (ESRD) patients receiving PD worldwide are approximately 200,000 and that they represent 11% of the total number of patients receiving any modality of dialysis (Jain et al., 2012). It is worth mentioning that Putman in 1923 made the first attempt for PD in a canine model (Putnam, 1923).

The extended use of PD over the years makes the in depth understanding of the physiology of the peritoneal membrane, the transport of solute and water across it and the mechanisms influencing inflammation, peritonitis, peritoneal injury and encapsulating peritonitis, a necessity. Moreover, many therapeutic interventions, new PD solutions, and pharmaceutical agents need to be tested before their implementation in the daily clinical practice. Research on humans could probably provide the necessary answers to the aforementioned topics, but it is complicated with many technical problems and ethical concerns (Stojimirovic et al., 2007). The scientific community in order to overcome this problem has exploited the observed similarities among humans and animal models. More precisely, the similar transport properties of solute and water across the peritoneal membrane in humans and animal models was an important foundation for the conduction of series of experiments in a variety of animal models.

Small animals such as rats, rabbits, and genetically modified mice have mostly been used for experimental models, although larger animals such as dogs, sheep, or even kangaroos have been used, as well. The use of each animal model comes with different advantages and disadvantages (Table 1). More precisely, rats are easy to breed and affordable, but have a short life expectancy, small size that increases the risk of complications during the insertion of a peritoneal catheter and large size of peritoneal membrane when at the same time only small quantities of dialysate can be introduced in their peritoneal cavity (Lameire et al., 1998). On the other hand, rabbits have a longer life expectancy, can survive longer on PD and the insertion of the peritoneal catheter is easier, but are very delicate animals and difficult to breed (Garosi and Di Paolo, 2001). Regarding larger animals, their life expectancy is long and could approximate PD on humans, but their breeding is difficult and expensive and a large time frame is needed for results to be obtained (Van Biesen et al., 2006).

**Table 1:** **Main advantages and disadvantages of different animals used in animal models**.

**Animals**		**Advantages**	**Disadvantages**
Small	Rats	Easy and affordable breeding Fast generation and maturation	Short life expectancy Small size—increased risk of complications Higher ratio of peritoneal surface area compared to humans Large size of parietal peritoneum High intraperitoneal amylase levels
	Rabbits	Adequate life expectancy Adequate survival on PD Easy peritoneal catheter insertion Similar size of parietal peritoneum to humans Similar ratio of peritoneal surface area to humans	Delicate animals—difficult to breed
	Genetically modified mice	Easy and affordable breeding Fast generation and maturation Ability to explore the role of single proteins	Extremely small size
Large	Dogs Sheep Kangaroos etc	Long life expectancy Easy peritoneal catheter insertion	Difficult and expensive breeding Large time frame for obtaining results

As for the genetically modified mice, both knockout and transgenic models have been used over the past few years in order to provide important information in many different molecular pathways that are significant for PD (Nishino et al., 2007). The ability to modify the genome of mice enabled the scientists to explore the expression of different genes and the results of the modification of their expression. The role of single proteins such as aquaporin-1 in the transport of water across the peritoneal membrane (Yang et al., 1999; Ni et al., 2006) or NOS (Nitric oxide synthase) isoforms in peritonitis (Ni et al., 2010), as well as, IL-6 in inflammation (Hurst et al., 2001; McLoughlin et al., 2004) and TGF-β in encapsulating peritonitis (Park et al., 2008) were explored using genetically modified mice. The extremely small size of these animal makes manipulations difficult (Ni et al., 2003), but over the past few years the development of new molecular techniques provide significant tools to overcome this disadvantage (Devuyst et al., 2010).

Acute and chronic animal models have been developed (Hoff, 2005; Pawlaczyk et al., 2015) (Table 2). Acute models describe experiments that have short duration and can study the impact of a single dwell on the peritoneal membrane. They provide crucial information regarding mainly the transport characteristics of the peritoneal membrane and its function. Its permeability to water and solute, the interaction with different dialysis solutions, certain substances, therapeutic agents or even inflammation mediators can be extensively studied this way. On the other hand, chronic models can evaluate the chronic impact of PD resembling the impact of the method on humans due to the longer duration of the experiments. They can focus on changes of the structure and the function of the peritoneal membrane. For example, the effect of glucose used as an osmotic agent in dialysis solutions or the effect of the newer biocompatible solutions with lower GDPs' (Glucose degradation products) concentration, neutral pH or replacement of glucose with icodextrin on the morphology of the peritoneal membrane can be analyzed. Fibrosis, encapsulating peritoneal sclerosis, repeated episodes of peritonitis, and morphological alterations can be in depth studied using the chronic models. In this way, an important background for targeted therapeutic interventions is provided.

**Table 2:** **The main studied parameters in acute and chronic animal models**.

**Animal models**	**Studied parameters**	
Acute	Transport characteristics and function of the PM	Permeability to water and solutes Lymphatic drainage Interaction with different dialysis solutions Acute effect of glucose Inflammatory response—effect of inflammation mediators Interaction with various therapeutic agents
Chronic	Changes of structure and function of the PM	Effect of biocompatible solutions (lower GDP and neutral pH) Effect of icodextrin Peritonitis—defense mechanisms Mesothelial cell damage PM thickness Vascular alterations Fibrosis Encapsulating peritoneal sclerosis Therapeutic interventions

*PM, peritoneal membrane*.

In the acute model the animal is anesthetized, a temporary catheter is inserted in the peritoneal cavity and during a 4-h dwell samplings of the peritoneal fluid and blood are obtained (Lameire et al., 1998). In the chronic model, on the other hand, three are the most common methods used (Mortier et al., 2005). The first method includes the direct blind injection of the dialysate into the peritoneal cavity with or without anesthesia. The repeated injections are related to increased risk of intraperitoneal bleeding and infection that could probably interfere with the results of the experiment. The second method is called “open system” and is characterized by the subcutaneous insertion of a permanent indwelling peritoneal catheter from the neck to the peritoneal cavity of the animal. This catheter can be used for the introduction of dialysate into the peritoneal cavity and the drain of the dwell directly or indirectly as it can be used as the tunnel through which another sterile catheter is inserted during every exchange. In this method anesthesia is not required but the rate of peritonitis episodes remains high and the malfunction of the catheter as a result of omental wrapping, adhesions and fibrosis is frequent. Finally, the third method is called “closed system.” In this method, as in the “open-system,” a permanent indwelling peritoneal catheter is inserted subcutaneously from the neck to the peritoneal cavity of the animal. The catheter is attached to a subcutaneous reservoir in the neck of the animal and the dialysate remains in the peritoneal cavity until it is fully absorbed. This method results in lower rates of peritonitis, although the malfunction of the catheter still remains a problem.

In any case, even if it is tempting, before applying the extrapolated results of animal models in humans we should point out the differences between these models and humans and the related technical problems. The most important differences concern the visceral, parietal and diaphragm surfaces. It seems that the diaphragm, which is directly related to the lymphatic drainage responsible for the clearance of macromolecules, extends to a larger area in humans than in animal models (Pawlaczyk et al., 2015). On the other hand, the parietal peritoneum that is directly related to the solute transport is larger in rats than in humans, while in rabbits it is similar to humans (Pawlaczyk et al., 1996). Moreover, it is known that the intraperitoneal levels of amylase that are elevated in rats can locally degrade icodextrin, an osmotic agent used in several dialysis fluids, rapidly (de Waart et al., 2001). To these differences one must add the higher ratio of peritoneal surface area to dialysate that is observed in rats, while in rabbits it is similar to the one observed in humans (Stojimirovic et al., 2007). Finally, in rats changes in the permeability and the surface area of the peritoneal membrane are encountered as they grow up, resulting in changes in the kinetics of the peritoneal membrane (Kuzlan et al., 1997). As Van Biesen et al highlights multiple experiments in different animal models and comparison of the results obtained could overcome those differences and provide a more realistic approach of the mechanisms that characterize PD in humans (Van Biesen et al., 2006).

Among the technical problems that have to be faced when handling animal models, tissue-sampling problems must be highlighted. The peritoneal tissue is quite fragile, dries quickly when exposed to the air and can easily be damaged even after delicate contact with surgical gloves or instruments (Duman and Sen, 2009). Furthermore, in patients on PD the visceral peritoneum exhibits more pronounced alterations than the parietal peritoneum, while in many of the studies conducted in animal models the biopsy samples are obtained by the parietal peritoneum. This inconsistency could be misleading if extrapolating results of animal studies to humans.

Catheter obstruction, peritonitis, and anesthesia use represent other major problems. In an attempt to minimize catheter dysfunction, omentectomy, and heparin have been used, but both methods have important pitfalls. On one hand the omentum is considered to be an important immune defense organ of the peritoneal cavity (Beelen, 1992), while on the other heparin is known to have many pleiotropic actions besides being an anticoagulant (De Vriese et al., 2001; Gozdzikiewicz et al., 2009). Therefore, both of these interventions could result in undesirable functional and morphological changes in the peritoneum (Mortier et al., 2005). The use of heparin coated catheters that has been suggested seems to be a good solution, since it reduces the rate of malfunction without inducing changes in the peritoneum (De Vriese et al., 2002). Regarding peritonitis, many investigators have administered antibiotics in order to prevent infections, an intervention that has been proven successful and simultaneously not leading to morphological and function changes in the peritoneum (Mortier et al., 2003; Choi et al., 2006). Finally, the use of anesthesia when handling animals has been related to changes in the peritoneum kinetics, probably through a direct reduction of the lymphatic drainage (Tran et al., 1993) and an increase of ultrafiltration and decrease of the absorption rates from the peritoneal membrane (Shin et al., 2006).

There are some important differences in animal model studies (Mortier et al., 2005; Topley, 2005). There are differences related to the volume of dialysate instilled and the time it remains in the peritoneal cavity, the frequency of the exchanges, and the total time the animals undergo PD before the termination of the study. The drainage of the dialysate is also an issue. More precisely, there are animal models characterized by the presence of the dialysate in the peritoneal cavity until it is fully absorbed, while in other animal models drainage after every dwell is performed. The last model resembles chronic PD in humans and offers the ability to better understand transportation through the peritoneal membrane and at the same time examine the dialysate for the presence of cells and substances (Pawlaczyk et al., 2015). Moreover, differences in the use of anesthesia, heparin or even the definition of peritonitis have been described. A common finding in most studies is the use of high concentration glucose solutions although in humans other lower glucose concentration solutions may also be used. Finally, the high rate of dropout due to catheter malfunction must be highlighted. It is obvious that there are many limitations when analyzing the results of the studies conducted on animal models, nevertheless the information they provide are very important and necessary to better understand this method of renal replacement therapy.

One field of special interest for nephrologists is encapsulating peritoneal sclerosis (EPS) which is a rare but life threatening complication of PD. Several animal models have been used in order to understand the underlying pathophysiological mechanisms that lead to EPS (Hoff, 2005; Park et al., 2008; Hirahara et al., 2015). Chlorexidine and acidic dialysis solutions, povidone iodine, formaldehyde, and even bleach and talc are some of the agents that have been used in an attempt to induce peritoneal injury and development of EPS in animal models. At the same time many different inhibitors of inflammation, angiogenesis, and fibrosis, as well as, inhibitors of the renin angiotensin system have been studied as potential therapeutic interventions in an attempt to resolve EPS (Kitamura et al., 2014).

The *in vivo* animal models provide useful information for future PD solutions that are yet to be tested or promote our understanding of their effects on the peritoneal membrane of ESRD patients undergoing PD. To this end an *ex vivo* sheep and human model of the peritoneal ionic and water permeability can further provide useful information that can be applied in the PD solution improvement and development area (Liakopoulos et al., 2006, 2009). In these studies the membrane specimens are mounted in the Ussing System that measures the transmembrane ionic permeability (Zarogiannis et al., 2004; Stefanidis et al., 2005). The majority of such investigations involved electrophysiological experiments in sheep visceral or parietal peritoneum specimens and some of the results have been tested in human specimens yielding identical findings, thus rendering this model a powerful tool for PD solution assessment as to the ion and water transport drug induced alterations (Zarogiannis et al., 2005, 2007; Stefanidis et al., 2007). The focus of these electrophysiological investigations was the identification of ion channels, as well as, hormonal receptors present in the mesothelial compartment of the peritoneum (Kourti et al., 2007, 2013; Zarogiannis et al., 2007; Karioti et al., 2008, 2009). Most of these findings remain yet to be investigated in human specimens and subsequently tested in *in vivo* animal models.

In conclusion, *in vivo* and *ex vivo* animal models have been extensively used for the understanding of the properties of the peritoneal membrane and results of these experiments have been extrapolated in humans. For the time being, the fundamental differences in the animal models used, as already described, remains the main problem when it comes to comparing results of different models and especially using these results in order to evaluate the use of PD in humans. At the same time, the necessity of in depth understanding of PD brings into perspective the importance of establishing standardized protocols that could overcome the aforementioned differences and obstacles.

## Conflict of interest statement

The authors declare that the research was conducted in the absence of any commercial or financial relationships that could be construed as a potential conflict of interest.
